# Shifting perspectives on the role of parents in the rehabilitation of children with higher body weight: insights from qualitative interviews with children, parents, and professionals

**DOI:** 10.3389/fped.2026.1797335

**Published:** 2026-03-24

**Authors:** Lucia Schlottner, Lucie Schröder, Sandra Fahrenkrog, Judith Stumm

**Affiliations:** Charité—Universitätsmedizin Berlin, Corporate Member of Freie Universität Berlin and Humboldt-Universität zu Berlin, Institute for Medical Sociology and Rehabilitation Studies, Berlin, Germany

**Keywords:** child and adolescent rehabilitation, higher body weight, outpatient rehabilitation, overweight/obesity, parental role, responsibility

## Abstract

**Introduction:**

Overweight and obesity are among the most common chronic diseases and are associated with physical and mental health consequences. Parents of overweight/obese children face additional psychological stress. The treatment of higher body weight should be multidisciplinary and multimodal and should involve parents as was implemented and evaluated in the outpatient rehabilitation program for children with higher body weight.

**Materials and methods:**

In the qualitative study arm of a controlled, two-arm, mixed-methods study evaluating the effectiveness of outpatient rehabilitation programs for children with higher body weight, the parental role within the rehabilitation program has been explored. Interviews with healthcare professionals and parents and focus groups with children and adolescents have been conducted and analyzed with the framework analysis.

**Results:**

The analysis of the interviews revealed that parents play a pivotal role in outpatient pediatric rehabilitation as motivators, role models, authority, and mediators in transferring therapeutic skills to daily life. While all groups—children, professionals, and parents—acknowledged the importance of parental involvement, differences emerged in role perceptions, concerning among others control and autonomy. Children expressed both appreciation and need for parental support, as well as a desire for greater self-determination in their parents’ involvement.

**Conclusion:**

Parents take on different roles in their children's rehabilitation. To enable them to fulfil these roles effectively, rehabilitation facilities should provide appropriate resources and offer individualized parent programs. A comprehensive concept for accompanying and supporting parents during their children's rehabilitation can clarify responsibilities and boundaries and reduce tensions between parents, children, and professionals.

## 1 Introduction

Overweight and obesity are among the most common chronic diseases and are associated with physical and mental health consequences. In Germany, 15.4% of children and adolescents^1^ between the ages of 3 and 17 are considered overweight, 5.9% obese, and 1% severely obese. Children with a low socioeconomic status are more frequently affected by obesity than children from families with a high socioeconomic status (1). Approximately 45% of obese children and 85% of obese adolescents remain obese in adulthood (2–4). Children with chronic conditions such as obesity are a vulnerable group from a health science perspective (5). They are dependent on their parents and are influenced by their decisions and behavior.

The families of chronically ill children are increasingly taking on an active and independent role in the care and, in particular, rehabilitation process (6). Klüpfel et al. shows that the stressors (e.g., parents' own health problems) and resources (e.g., access to education/health literacy, stable social environment) of parents are not sufficiently assessed before the start of therapy. This leads to misunderstandings between medical/therapeutic professionals and parents and, in the long term, to insufficiently sustainable therapeutic success for children (7, 8). Parents of overweight/obese children face additional psychological stress. In addition to their children's health limitations and risk factors, they often suffer from socially normalized aesthetic ideals of the body, which manifest themselves in discriminatory and stigmatizing behavior toward overweight/obese children and adolescents in everyday life (9–11). It is not uncommon for obesity to already exist in the family and for parents to have experienced discrimination themselves (12, 13). Children and adolescents who are overweight or obese and their families often face social disadvantages (6). It can be assumed that this is a vulnerable group of people who suffer multiple forms of discrimination (14). It is therefore particularly important to take a closer look at the individual living environments and everyday circumstances of parents and to adapt a parent program to the everyday circumstances of families in a low-threshold manner.

The biopsychosocial model for the development of overweight/obesity in childhood and adolescence takes into account biological, genetic, psychological, and social factors that play a role in its development, change, and management. Therefore, the treatment of obesity should be multidisciplinary and multimodal (15). In this context, outpatient care programs provide low-threshold, everyday support (16).

The family and social context is particularly relevant and influences the sustainable behavioral change of children. In their function as role models, parents have a major influence on the motivation and behavior of their children through their motivation, participation in the program, and long-term changes in the home environment. The individual living environments, resources, possible health impairments, and other stresses on families also influence cooperation with parents and are difficult to compensate for in the context of an outpatient rehabilitation program (8).

Targeted and individual changes to the living environments of children and adolescents and their families are considered the most important measure (2). The involvement of parents in therapy is therefore essential. Success can only be achieved and the parent-child relationship strengthened by motivating the whole family and addressing the children's and adolescents' lifestyles, habits, and attitudes (17).

The following article is based on the results of the qualitative study arm focusing on the parental role and involvement of their children's therapy of a project funded by the German Federal Pension Insurance (Deutsche Rentenversicherung Bund) Fund to evaluate an outpatient rehabilitation program for children with higher body weight.

The evaluated programme is being implemented in four rehabilitation centres in Germany and is aimed at children aged 6–18 who have been diagnosed with overweight or obesity. One parent takes on the role of co-rehabilitation partner during a 13-week treatment period. Children and their parents participate in an initial intensive week lasting five full days, completing therapy sessions together. In the following twelve-week school-accompanying phase, children come to the rehabilitation center two afternoons a week and attend group courses. From the second week onwards, parents take part in program activities and training courses once a week. Both the children and their parents are supported by a multidisciplinary team throughout the entire period.

This article focuses on qualitative data collection in the context of process evaluation. The research was undertaken as part of a postdoctoral grant funded by the Wilhelm Foundation, which focuses on understanding parental influence and involvement in pediatric rehabilitation.

The following question will be answered below: What roles and role expectations are attributed to parents in outpatient pediatric rehabilitation by children, professionals, and the parents themselves, and how do these perspectives differ from one another?

## 2 Disclaimer

The German medical system categorizes individuals as underweight, normal weight, overweight, or obese. Following Schröder et al. (18), these categories may be perceived as discriminatory. The present study adopts the term “higher body weight” to encourage a more sensitive and non-stigmatizing use of language. When referring to other studies, the original terminology—in some cases “overweight” or “obesity”—is retained, consistent with the respective authors' usage.

## 3 Materials and methods

This article presents findings from the EvambAdi Study (Evaluation of an outpatient rehabilitation programme for children and adolescents with obesity), a controlled, two-arm, mixed-methods research designed to evaluate the effectiveness of outpatient rehabilitation programs for children with higher body weight. The overall study consisted of questionnaires administered at the beginning and end of the rehabilitation program, as well as six months after its completion. Participating children and parents also completed audio and written diaries at four different points during the rehabilitation period. In addition, a process evaluation was conducted, which included interviews with professionals, interviews with parents, and focus groups with participating children and adolescents. The outcome and process evaluations were conducted in parallel.

This article focuses on the interviews with healthcare professionals and parents as well as focus groups with children and adolescents undergoing the rehabilitation program.

Using a mixed deductive and inductive approach, we analyzed data from the EvambAdi Study to explore and characterize the multifaceted role of parents within these rehabilitation programs. The qualitative methodology was selected to capture the subjective experiences and diverse perspectives of study participants regarding parental involvement and participation.

Funding for this study was provided by the German Pension Insurance Association (Grant number: 8011-106-31/31.27.30), with no role in study design or content. A positive vote for the implementation of the project was given on 27th July 2023 by the Charité Ethics Committee (EA2_059_23). The COREQ checklist (COnsolidated criteria for REporting Qualitative research) (19) is used to describe the methodological approach (Supplementary Appendix 1). The following section draws on methodological explanations from previous publications in the course of the EvambAdi study (8, 18).

### 3.1 Sample and recruitment

All four rehabilitation centers are outpatient rehabilitation centers, located in Germay, that provide rehabilitation for both children and adults. The cooperating centers applied the same program in terms of its structure and design, which could vary slightly in its specific implementation, for example due to location factors, staffing, and financial and material resources.

Three distinct participant groups were recruited for the study: children, healthcare professionals, and parents. The participating children had all completed an outpatient rehabilitation program. Eligibility criteria for the children included an age of 6–18 years, a weight percentile above 90th percentile, and fluency in German. Exclusion criteria were a BMI below the 90th percentile, lack of German language skills, and age below 12 or above 18 years. and early termination or incomplete participation in the rehabilitation programme. Parents of these children were additionally recruited.

The healthcare professionals interviewed had at least one year of professional experience in medical pediatric rehabilitation. The aim was to include the perspectives of as many professional groups as possible who are involved in the rehabilitation program. The professionals interviewed were deliberately selected by the respective coordinating or managing employees of the relevant rehabilitation centers in order to recruit a heterogeneous group of participants in terms of age, gender, specialist discipline, and professional experience. Appointments were also coordinated by the respective senior staff members. All staff members at the rehabilitation centers who were asked agreed to participate in an interview.

Before the interview began, the interview participants were informed about the study both in writing and verbally and gave their written consent to the collection of data in anonymized form. None of the participants refused to be interviewed.

### 3.2 Research team

The research team consisted entirely of female researchers with diverse academic backgrounds. LuS is a master's student in social sciences at Humboldt University of Berlin; LS is a medical student and doctoral researcher at Charité University Medicine Berlin; and JS is a postdoctoral health scientist specializing in qualitative research and served as the project supervisor. The team was further supported by additional researchers, including SF, who contributed extensive expertise in qualitative methodology.

### 3.3 Guide creation and data collection

The guidelines (Supplementary Appendix 2) were developed by the study team in an iterative process and adapted and applied openly in the course of the study. The approach of Helfferich et al. and the 4-step formula “SPSS” (collect, check, sort, summarize) served as methodological guidance (20). The main topics were the rehabilitation program, cooperation with the children and adolescents and their parents, optimization of the program, and weight discrimination. In a first step, the guide was piloted and adapted with medical professionals from our own research institute. After piloting the first interview with professionals from the rehabilitation facilities and in the course of the study, minor changes were made to the content and structure of the guide to improve precision.

Between August and November 2023, 19 telephone interviews were conducted with healthcare professionals. Parental telephone interviews (*n* = 19) were carried out between October 2023 and December 2024, and six focus groups with 35 children (three to seven children per group) between October 2023 and January 2025. The interviews lasted an average of 50 min (range: 22–95). They were mainly conducted by LS as part of a doctoral thesis. Face-to-face interviews could not be held due to the geographical distances between the four different rehabilitation facilities and the limited availability of medical professionals. The focus groups with the children were jointly moderated by LS and JS on site in group rooms already familiar to the participating children. Only researchers and participants were present. With a total of 38 interviews and a total of 35 children and young people participating in the focus groups, content saturation is assumed. During the interviews and focus groups, memos were taken and these were taken into account in the analysis. No interview or focus group has been repeated.

There were no existing personal or professional relationships between the researchers and participants prior to the study. Participants were informed that all researchers were affiliated with Charité University Medicine Berlin and that there was considerable research interest in the topic. The location of the interviewees at the time was not asked.

### 3.4 Data analysis

The interviews were recorded in digital audio format and transcribed in accordance with the simplified transcription rules according to Dresing and Pehl (21). The interviewees did not subsequently have access to the transcripts of their interviews, meaning that they were unable to provide feedback on the results. The evaluation was carried out using content analysis with framework analysis (22) in the research team, combining deductive and inductive methods with the support of the MAXQDA2024 software program (VERBI Software GmbH, Germany). The deductively and inductively derived categories and codes were derived from the researchers’ (mainly by LuS and JS) theoretical preconceptions and assumptions, as well as from the interaction in the research context (Supplementary Appendix 3). Framework analysis is not subject to any theoretical approach. Rather, it is a flexible tool that aims to generate themes and can be adapted to various qualitative approaches.

## 4 Results

**Figure 1:**
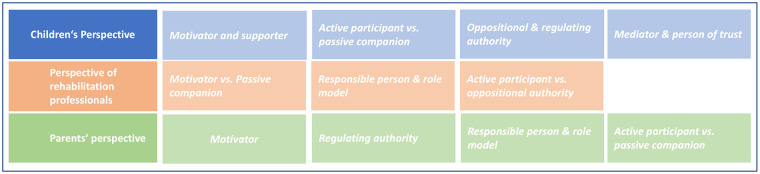
Overview of topics from different perspectives.

Six focus groups were held with a total of 35 children, along with 19 interviews with healthcare professionals and 19 parents (Supplementary Appendix 4). The analysis of the interviews revealed multiple themes addressing the impact and importance of parents within outpatient pediatric rehabilitation: parental roles and responsibilities, parent-child-relationship, challenges and the need for external support. We gained deeper knowledge about role expectations and perceptions from the perspective of children, professionals, and parents themselves. We compared the appearance and description of parental roles (see Figure 1 for topic overview).

The quotations have been smoothed for better readability and translated from German into English.

### 4.1 Children's perspective

#### 4.1.1 Motivator and supporter

Descriptions from focus groups reveal that parents should foster their children's motivation by positive behavior, emotional support, and active participation in health-promoting activities such as physical exercise and balanced diet. In addition to practical help—such as participating in joint activities—children highlight the emotional support they receive from their parents.

In particular, they identify their mothers as a central supporting role. The physical presence and commitment of parents during rehabilitation are perceived as motivating. On the other hand, absence or lack of support from parents can be experienced as demotivating. Parental behavior is perceived ambivalently. It can have both a supportive and an inhibiting effect, depending on the form of involvement and the quality of communication.

“(…) I'm also doing it a little bit for my mom, because she wanted us to make a change.” (Reha2_FG3)

“(…) I also think it's good that my mother is involved in everything here, so that we can do it as a whole family at home and I don't feel like I have to do it alone. Because we're all on the same page (…).” (Reha1_FG6)

“B1: When we have rehab on Tuesday and are supposed to do sports together. I think that's really stupid.”

I2: Why?

B1: Because then I'll be sitting there alone.

I2: Even though your mother is there?

B1: Yes.

I2: But…Oh, because she can't participate because…

B1: Yes, because she has a belly.

I2: Ahh, okay. But would you like it if (…) she (…) could join in…

“B1: Yes.” (Rehab2_FG4)

### 4.1.2 Active participant vs. passive companion

In the focus group discussions, children describe their parents as both active participants and passive companions during rehabilitation. Active parental involvement is viewed as essential. It strengthens the bond and helps parents gain practical knowledge and skills through shared experiences. In contrast, passive behavior is seen as frustrating and demotivating. Genuine engagement is described as important for emotional and practical support.

Joint learning and participation are experienced by children as connecting and supportive. When parents are actively involved, they experience the content directly. Children explain that it prevents them from having to pass on all information at home. However, conflicts may occur, for example through parental control, arguments, or perceived unfairness—such as when parents are allowed to rest, but children are not. Parental presence is judged negatively if it remains too passive. In addition, according to the children parental presence can reduce their openness, especially regarding personal or stressful topics.

“Well, I think it's practical because you don't have to come home and tell your parents everything that happened today. And we don't have to implement it on our own at home because our parents don't understand what we mean. This way, they are involved and know what's going on themselves (…).” (Reha1_FG2)

“(…) adults are allowed to say, ‘I can't do it anymore,’ they're allowed to sit down, and the children have to carry on. And that's just one of those things that I think is stupid.” (Reha2_FG3)

“(…) if I talk about personal problems, for example about mental health or something like that, then I can have my mother there, (…) but I prefer to talk about it in private because I don't want to burden my mother with it.” (Reha1_FG6)

#### 4.1.3 Oppositional & regulating authority

Some children describe their parents as an oppositional authority that sends contradictory or discouraging signals. On the one hand, it is outlined that parents demand weight reduction or behavioral change. On the other hand, they make negative comparisons or insulting remarks, which undermines motivation. Some children mention that parents show rejection toward participation or the rehabilitation goals.

According to the children, a lack of parental support and willingness to change leads to feelings of hopelessness or being left alone in the process. Some of the children wish for more self-determination regarding the role of their parents, for example in the rehabilitation setting or during medical examinations.

Parents are also described as a regulating authority that sets rules and controls behavior. At home, this may happen through fixed structures such as mealtimes or dietary rules. From the children's perspective, parents take on monitoring functions during rehabilitation, for instance by checking whether exercise routines are performed correctly. This kind of supervision often leads to annoyance or resistance among the children, especially when it is perceived as overly controlling.

“I don't know, my father is the kind of person who uses the fact that I'm attending this program against me. My parents generally just want me to lose weight. But at home, I get comments or harsh criticism (…).” (Reha1_FG6)

“But I find it worse when my parents are in the room, because my parents are really the kind of people who ask me all the time: ‘So how much do you weigh now, how much now?’ And that's really annoying, because I find it very uncomfortable. And even if I tell them, they keep doing it. That's why I don't want them to be in the same room when I'm being weighed. (…) And that's not really motivating.” (Reha1_FG6)

“Well, I really didn't have freedom of expression. Mmh, bam, we're going there.” (Reha1_FG2)

“I1: (…) you come home from school and then you come to rehabilitation center and then you probably don't get home until 7 p.m. or later, right?

B3: Yes, and after that I'm not allowed to eat anything.

I2: Okay, because that's what they say in rehabilitation center?

I1: No, at home…

(…)

I1: And can you talk about it, or have you tried talking about it?

“B3: Not really. Well, you can imagine it like this: I couldn't tell a bull, “Don't run at me.” My father is relatively stubborn; when he hears something, you could imagine him like a wall that just won't let you through.” (Reha1_FG2)

#### 4.1.4 Mediator & person of trust

Parents may act as mediators between children and professionals. They provide an important basis of trust, and mothers are most often mentioned as central figures. Some children describe that mediation by parents can create a sense of security, especially during medical examinations.

“I think if you have to be weighed, maybe your guardian should do it (…) And they could just tell the doctor without telling you, so (…) you don't have to hear it. Because for some people, the problem is that (…) they start blaming themselves.” (Reha1_FG6)

“Well, I tell my mom almost everything because I can talk to her about anything. I just trust my mom. Of course I tell her, and then we'll find a solution to this problem (…).” (Reha1_FG5)

“I wish that no one except my doctor and my mother will know my weight, and when I die, nobody will find out. Haha.” (Reha1_FG5)

### 4.2 Perspective of rehabilitation professionals

#### 4.2.1 Motivator vs. passive companion

In the interviews, rehabilitation professionals distinguish between motivated and non-motivated (or passive) parents. Parents are expected by health professionals to enter rehabilitation with their own motivation and initiative. They should act as a source of motivation for their children, provide active support, and encourage them throughout the rehabilitation process. Parents are regarded as a decisive resource for the success of rehabilitation. An active attitude on their part is expected to promote the long-term effectiveness of what has been learned. Parents are also expected to recognize the necessity of their own participation.

So described “passive” parents, on the other hand, show little or no involvement and do not take part in the activities offered. According to professionals, these parents see their role mainly as accompanying their children rather than actively contributing. Some parents are perceived to expect the professionals to “solve” their children's problems. Rehabilitation is therefore sometimes anticipated as a form of delegating the problem.

It is also observed that parents' motivation, in the perspective of health professionals, tends to decrease over the course of rehabilitation. According to the professionals, they can partly compensate for a lack of motivation among children, but they are not able to do so in the case of parents.

“So one big challenge is actually motivating the participants. They have varying levels of motivation (…).” (Reha2_I11)

“(…) the two main headaches are that so few parents have the motivation to tackle this. (…) Oh, I sometimes really think I might as well have talked to the wallpaper. Because it very often doesn't get through. They are so far removed from their feelings sometimes, and the motivation is there at the beginning, but it ebbs away for most of them. (…)” (Reha2_I16)

“I think working with parents is very important because they have to support the project and stand behind it. Children can't do it alone, and often parents don't realize that it's not enough to just send their child to rehabilitation center, it's a task that involves much, much more.” (Reha3_I14)

“(…) I would say that you may have encountered one or two parents who give you the impression ‘I want my child to solve this problem and I want to be as uninvolved as possible. I have my own issues to deal with,’ and that can sometimes be a bit difficult (…).” (Reha2_I2)

#### 4.2.2 Active participant vs. oppositional authority

The active participation of parents is considered essential by professionals for the effectiveness and sustainability of rehabilitation. According to them, it is necessary to see parents explicitly as participants and parents themselves must also accept this role to ensure the success of rehabilitation. Professionals aim to keep parents “on board” throughout the process to create long-term stability in therapy. Work with parents is regarded as a central element of outpatient rehabilitation. Regular parental participation is supposed to strengthen children's motivation, supports mutual understanding, and creates shared experiences, often described as a “family project.”

Professionals expect supportive and open behavior from parents that contributes to changes in the home environment. Parents should act as contributors who actively participate in the process of change rather than slow it down or block it. It is observed by the professionals interviewed that some parents only realize at the start of rehabilitation that they are expected to take part and contribute. Therefore, professionals express the wish for greater parental involvement and for a clearer definition of their role as active participants.

Some parents are seen as counterproductive, especially when compared to the high level of engagement shown by the professionals. While professionals perceive themselves as initiating change and “getting things moving”, parents are seen as slowing down or resisting these processes. It is suggested that some parents do not perceive a need for change or consciously avoid making changes. The partial “failure” of rehabilitation is sometimes attributed to the lack of parental cooperation.

Professionals acknowledge that parents often have low motivation after long working days. However, they compare this with the children who also attend school. Within family dynamics, the less involved parent—often separated or less engaged—is described as an oppositional figure.

“It's often the case that parents only realize [during the intensive week] that this isn't just fun and games, they have to do something, it only becomes clear during the program.” (Reha2_I12)

“(…) so when we have parent-child activities, we always see the same parents who take the time to come, who help with the cooking, who join in the movement games. And there are parents who don't come. Because they have to work, because they don't have the time.” (Reha3_I7)

“Every now and then you hear rumors that it often fails because the parents don't go along with it. For example, the father doesn't participate and still buys thick slices of salami and all sorts of other things, and no attention is paid to nutrition or to ensuring a high proportion of vegetables. (…)” (Reha1_I13)

#### 4.2.3 Responsible person & role model

Parents are expected to actively assume and fulfill their responsibility. From the perspective of the health professionals, parents share responsibility for the success of rehabilitation and help shape the process. In this role description, parents also serve as key role models for their children—in eating behavior, daily routines, and parenting practices. The interviewees point out that children orient themselves toward their parents' behavior, routines, language, and attitudes. Since doctors and other professionals can only provide guidance, responsibility and motivation may lie primarily with the parents.

The interviewees express that parents are expected to take responsibility for daily life, nutrition, and routines. Furthermore, health professionals mention the goal to encourage active parental involvement despite external pressures such as work, time constraints, and limited resources.

Professionals note that many parents find it difficult to fully accept and actively implement their responsibilities. Everyday stress is seen as a factor that limits their ability to act responsibly. In contrast, positive parental role models have a motivating effect on other families within the rehabilitation group.

“(…) I think parents like to pass on the task or responsibility. (…) They expect a lot from US and are rarely willing to change anything themselves. (…) THEY are the ones responsible and have to limit the time, because the CHILD cannot limit it.” (Reha2_I16)

“(…) but they just can't seem to get off their butts and say, ‘Come on, let's sign up for this, let's do it,’ and just take responsibility. I think that's what's missing too much. And sometimes I get really frustrated after a day at the children's rehabilitation center (…)” (Reha2_I16)

“(…) when we look at the parents (…), we can see very clear parallels, and in fact, the parents are almost always overweight too. (…) But this is actually a home-grown problem, because it may be modeled by the parents or lived by them.” (Reha1_I3)

“Of course, children also bear responsibility for themselves depending on their age, but they learn this from their parents or grandparents; they have to learn it. If it is not modeled for them, it is difficult.” (Reha3_I14)

### 4.3 Parents' perspective

#### 4.3.1 Motivator

The motivation of their own child is at the core of parents' understanding of their role. To achieve this, parents describe using various strategies such as participating in rehabilitation together, applying psychological tools learned during the program, strengthening their child's self-esteem through praise, and establishing shared routines.

Parents report that they often must take over the motivation for “going there” on behalf of their children, which they sometimes find more challenging than the actual participation itself. They also anticipate that their children will perceive them as a source of support. However, according to their accounts, parents experience phases of low motivation during rehabilitation—both individually and together with their children.

“The first few weeks were great, it was well received, not only by my child, but also by me. But at the moment I'm in a bit of a slump, because 12 weeks is a damn long time. And I keep thinking to myself, ‘Phew, that's enough.’ (..)” (Reha1_I4)

“Well, it's pretty stressful sometimes (laughs) when you come home from work and then have to organize everything. (…) But you know what you're doing it for. We're learning something. And I can't motivate [child's name] as much on my own as they do in rehabilitation center (laughs).” (Reha1_I13)

#### 4.3.2 Active participant vs. passive companion

Parents largely see themselves as active participants. Joint activities and everyday routines—such as cooking, shopping, and exercising—are experienced as essential for the effectiveness and sustainability of rehabilitation. Among other goals, parents aim not to attribute difficulties solely to the child but to recognize and address them as a shared family issue. They also strive for improved communication, joy in shared successes, closer relationships, and a stronger sense of teamwork through participation. Some parents describe their participation as a team partnership. According to them, the success and sustainability of change depend on the active involvement of the entire family, which they refer to as the “family project.”

Parents take on their own tasks and are willing to contribute. The active role is often perceived as meaningful and helpful. The combination of joint and separate sessions is viewed positively. Encouraged by the rehabilitation process, parents postulate to increasingly understand difficulties as shared problems rather than as the child's deficits, even though this perspective is sometimes challenged by everyday situations. Through shared activities and mutual support, parents describe a developing sense of “we,” which may strengthen the parent–child relationship. The implementation of joint activities—especially in everyday areas such as cooking and exercise—is considered crucial for success when approached as a team. This self-perception as participants or team partners also appears in how parents anticipate their children's view on their roles.

“(…) but what's much more important is that it's not just the child who is taught or given tools to deal with this, but also the parents. (…)” (Reha1_I1)

“(…) And I wanted my daughter to see and recognize WHERE the problems [are], or what we need to work on, because it's not just mine or not just [my daughter's] problem, it's more of a joint effort, a family matter, a change. And we should all work on it together.” (Reha1_I2)

In contrast, some parents illustrate themselves mainly as companions who “run alongside” the process. They view rehabilitation as something meant for the child. As a result, they express a wish not to attend all weekly sessions. They also emphasize that as children grow older, their need for independence increases, and parental influence naturally becomes more limited. The children are seen as independent enough to take part in rehabilitation on their own. Professionals are described as focusing on the child and individual needs.

“My child has to go twice a week and I only have to go once. I think every other time would be enough for one parent. Because it's not about the parent, it's about the child (…) now after 12 weeks. So I say to myself: ‘Yes, now the energy has gone’ and I'm glad that from next week we only have to come a few times. So I think a shorter period makes more sense for the parents.” (Reha1_I4)

“I think a clear distinction is important, because once they reach a certain age, you have no influence on them.” (Reha1_I11)

#### 4.3.3 Responsible person & role model

Parents see themselves as the primary authority responsible for health, nutrition, and changes in family routines. They consider it their duty to guide and lead their children toward healthy behavior. This role is partly justified by the idea that adults are more aware of health risks than children, who cannot yet assess them adequately. For this reason, parents see it as their—or in some cases the doctors—obligation to take responsibility.

Responsibility is understood as a modeling and steering function, since children cannot implement behavioral changes on their own. Parents describe the perception that their own behavior strongly shapes their children's attitudes and health habits. They report that their children imitate what they do. This modeling effect is also highlighted when discussing how children are assumed to perceive the parents' role.

In rehabilitation, according to the interviewed parents responsibility is addressed as a topic in a supportive and solution-oriented way, without assigning blame. Rehabilitation is experienced as an impulse for self-reflection, during which parents recognize their own “blind spots” and learn to take responsibility actively. It is also characterized as a “wake-up call” that increases awareness of their active role in the process of change. Parents describe that therapists are available for questions, but parents are expected to take responsibility for seeking help when needed. Shared activities are seen as motivating and strengthen the sense of togetherness. Parents brand the rehabilitation program as encouraging them to recognize and communicate their role-model function more consciously.

Parents experience joint activities during rehabilitation as motivating, yet everyday life is often marked by setbacks and the challenge of balancing obligations with their role-model function. Parents reflect that their own eating habits are strongly shaped by what they were taught as children. Change therefore requires adaptation of personal routines and attitudes.

A tension exists between parental responsibility and social expectations, which becomes particularly evident during rehabilitation. Parents sometimes reflect critically on their own actions and feel guilty when change does not succeed or when higher body weight is viewed as a parental failure. A similar tension is seen in fulfilling their role as models, positioned between parental engagement, the child's own motivation, and external social pressures. Parents also point to the limits of their role-model function, as children must develop their own responsibility, and societal constraints can make consistent implementation difficult.

“It wasn't like they said, ‘It's your fault’ or anything like that; that didn't come up in rehabilitation center. But it did strengthen us as parents. It wasn't dismissive, but really focused on, ‘What can we do better or differently, or where can we improve?’” (Reha2_I12)

“(…) The parents themselves were also shaken up a bit, I would say, in the sense that we actually have more responsibility than we thought. (…).” (Reha2_I14)

“(…) And it has to fit into my life, right? I can't just say: Okay, I'm going to devote 150% of my time to making sure my child is slim, cooking super-healthy vegan, vegetarian meals every day so that he really has NOTHING fatty, doing three hours of sport with him every day. I can't do that, my life doesn't allow it (short laugh). (…)” (Reha2_I19)

“She is open to new things, but of course she still falls back into old habits from time to time, and so do we, we have been setting an example for her all her life.” (Reha1_I11)

“(…) when I set an example, she sees that I also do sports, for example. She sees ‘Mom does sports, so why shouldn't I exercise too?’. Together, we function as a team.” (Reha2_I12)

#### 4.3.4 Regulating authority

Parents also assign themselves the roles of guidance and regulation. This becomes clear in the context of eating behavior, such as controlling portion sizes and selecting meals. From the parents' perspective, this regulatory function is necessary to ensure that their children's eating habits support good health. At the same time, children are encouraged to develop a sense of self-responsibility and to make more of their own decisions. Parents understand their role as a form of family management that must balance control and freedom.

In practice, parental control varies according to parents' accounts. In some cases, children accept parental regulation, while in others, conflicts arise because of these controlling actions. Parents point out a dilemma: on the one hand, they are expected to allow freedom and promote independent behavior; on the other hand, they are tasked with supervising eating habits. These expectations are not always easy to reconcile.

“(…) Because a child of that age is not capable of shopping for themselves in order to cook. So I blame myself for the fact that [child's pronoun] is now overweight. Because I think to myself: ‘Okay, I should have been stricter, I should have enforced something more, I should have forbidden things’.” (Reha1_I10)

“(…) And you want to give the child, the teenager, headspace, (…), but when it comes to eating, you should control them. At some point, it becomes difficult as an adult to intervene.” (Reha1_I4)

“Well, nutrition is mostly up to me, the parents. When I cook, we eat what I cook, so the change has to happen in my head. Sometimes it works out better, sometimes not so well (light laughter).” (Reha2_I18)

## 5 Discussion

Qualitative interviews with professionals from rehabilitation centers and parents, as well as focus groups with children and adolescents with higher body weight who are undergoing rehabilitation, were used to explore perceptions of the role and responsibilities of parents in the rehabilitation process and beyond from different perspectives.

Children, parents and specialists alike agree on the key role played by parents as central motivators and “key figures’ in transferring skills to everyday life. Parents' participation in the outpatient rehabilitation program is not questioned but rather demanded.

The responsibility that parents bear and their part as role models is increasingly emphasized by parents and professionals, and this is also implicitly reflected in the children's statements, in which they see their parents as mediators and perceive them as providing a great source of support. While parents assign themselves a regulatory and controlling function (and so do their children), professionals tend to describe these tasks as part of the necessary assumption of responsibility.

Nevertheless, some differences in perception can be identified from various perspectives. From the children's perspective, for example, an ambivalent attitude can be observed. On the one hand, their parents are motivators for them. On the other hand, they also take an oppositional position. They view their parents' active involvement in the rehabilitation program positively, but at the same time they criticize their parents' sometimes passive presence, the control of exercise and their dismissive comments. The focus groups suggest that the children would like to have a certain degree of self-determination regarding their parents' role and presence.

In addition to many advantages, family-based programs also cause conflicts and friction within families. Children often receive little to no support from their parents, who fail to change their unhealthy lifestyles. This can be perceived as intra-familial stigmatization (23). Whereas extant literature has extensively examined weight stigma in societal, peer contexts and in the health system (19), the manifestation of stigma within familial settings has received comparatively limited attention. Stigmatization by family members can be more hurtful and have psychosocial consequences. This can be difficult for professionals to identify, as it takes place outside the therapy program, in the patient's own home. It is therefore recommended that healthcare professionals should ask parents and children about any conflicts arising from their family intervention participation. To facilitate this, it may help to emphasize two key points: first, changing established habits is challenging for everyone, and second most children require additional motivation, with parents playing a critical supportive role (23).

The health professionals distinguish between “motivated” and “passive” parents. In their opinion, active parents are a prerequisite for successful rehabilitation, while passive parents tend to be a risk or a hindering factor. They also emphasize the role of parents as co-responsible and as role models. Overall, a kind of blame, if only implicit, can be discerned in the focus on parental responsibility. This is also confirmed by the article by Stumm et al. from the same project. According to the professional staff at the rehabilitation facilities, the passive role of parents poses a challenge for the implementation of the outpatient rehabilitation program and has an impact on the long-term success of the program (8). According to Smith and Samuels, the passive and active roles can be understood as a continuum. The passive or active role that parents assume depends, among other things, on the type of intervention in which parents participate and on how much responsibility the professionals assume in each case. So far, professionals seem to have little understanding of the different roles assumed by parents, and their capacity and resources to support them are limited (24).

Structural stressors as a cause of parental “behavior” tend to be neglected by professionals. The scoping review by Smith and Samuels identifies various roles of parents in the context of their children's rehabilitation. The authors recommend closer examination of individual and contextual factors that influence parental roles, such as stress and self-efficacy (24).

Given the shortcomings in the care provided to children with higher body weight, the role of parents is important not only in the context of rehabilitation itself, but also beyond. Joisten et al. highlight the urgent need for structural improvements in the care of children and adolescents with obesity based on the results of their survey of pediatricians. 82% of 802 pediatricians surveyed cited lack of time and limited infrastructure as causes of difficulties in obesity care (25). Strengthening the role of parents in the treatment of childhood obesity could empower parents, regardless of structural conditions and support from pediatricians, to sustainably implement the information learned in rehabilitation and the behaviors learned in everyday life.

In a pilot study by Ausner and Hampel, additional motivation-based family coaching was accepted by both children and accompanying parents in obesity rehabilitation for children between the ages of 10 and 17 and was rated as good to very good. The aim was to use motivational interviewing to improve both the children's personal responsibility and health behavior and the parents' personal responsibility, support for health management and parenting behavior (26). The results from qualitative surveys from the same project suggest that the objectives of family coaching could be strengthened for both parents and children (27). This reinforces the findings of the article by Klüpfel et al., which recommends the further development of companion programs in pediatric rehabilitation (7).

An expansion of family coaching for appropriate aftercare programs or for transfer into everyday life would certainly be recommended.

King et al. propose a framework concept for family-oriented interventions. Key aspects of the framework are, on the one hand, focusing on the needs of parents and expending capacities and, on the other hand, creating services for parents, such as information sources, self-help groups and psychosocial support services. Combined parent-child services are an important way to meet the needs of all family members (28).

## 6 Conclusion

Parents take on different roles in their children's rehabilitation, both consciously and unconsciously. The roles they take on and how they fulfill them depend on various family and personal contextual factors, such as resources, stress levels, and their relationship with the child. In addition, the roles are perceived, valued and interpreted differently by the children and the professionals as well as by the parents themselves, although all the participants interviewed agree on the necessity and importance of involving parents in their children's rehabilitation.

In the rehabilitation of children and adolescents with higher body weight, parents ideally take on an active role as motivators for initiating rehabilitation, as persons of trust and role models for their children, and especially in transferring what has been learned in rehabilitation into everyday life. For this reason, it is essential to empower parents to take on a supportive role in the rehabilitation process. The involvement of parents in the rehabilitation of children should be as individualized as possible in order to take personal and family context factors into account. Such a comprehensive concept for accompanying and supporting parents during their children's rehabilitation could also define responsibilities and boundaries, thereby reducing tensions and misunderstandings between parents and professionals, as well as between parents and children.

### 6.1 Strength and limitations

The strength of the study lies in the high number of interviews and focus groups that were conducted. This allowed for a diversity of perspectives to be presented and saturation to be assumed. Since the interviewees were selected by coordinators from the rehabilitation centers, it can be assumed that mainly motivated and reflective parents and rehabilitation staff took part in the interviews, thus creating a selective bias. It would certainly have been interesting to record the parents’ level of education and average income. This would have allowed us to draw conclusions about the possible presence or absence of resources in the families. Since overweight and obesity can also have a potential influence on the success of rehabilitation and on the parent-child relationship, measuring the parents' BMI and their own perception of their body and weight could provide additional insights. Reference could also be made to health information or pre-existing conditions in this context for other family members such as siblings and grandparents.

## Data Availability

The datasets presented in this article are not readily available because for data protection reasons, the data set is not available to the general public. Only study staff have access to the anonymized data. Requests to access the datasets should be directed to judith.stumm@charite.de.

## Footnotes

^1^For reasons of clarity and readability, the term “children” is used throughout the manuscript to refer to both children and adolescents.

## Supplementary material

The Supplementary Materialfor this article can be found online at: https://www.frontiersin.org/articles/10.3389/fped.2026.1797335/full#supplementary-material
