# Supplementary material for: Shifting perspectives on the role of parents in the rehabilitation of children with higher body weight: insights from qualitative interviews with children, parents, and professionals
Source: Front Pediatr. 2026 Mar 24;14:1797335. doi: 10.3389/fped.2026.1797335 (PMC13055526; doi:10.3389/fped.2026.1797335)
Supplement: Supplementary file 2 [file Table2.docx]

# Appendix 2: Interview guidelines

# Children and Adolescents Focus Group Guide

| Theme | Main Question | Follow-up Question | Special Methods | Remarks |
| --- | --- | --- | --- | --- |
| **Icebreaker / Introduction** | | | | |
| Introduction Round | Each child shares their name, age, and what they like to do in their free time |  |  |  |
| **Main Part** | | | | |
| **Theme: Rehabilitation Program** | | | | |
|  | Great, now let's start with the questions… |  |  |  |
|  | How do you feel here at the rehabilitation program? |  |  |  |
|  |  | Do you feel comfortable or uncomfortable? |  | Why? |
| Motivation | \| Why did you join the rehabilitation program? \| \| --- \|  \|  \| \| --- \| |  |  |  |
|  | For whom are you participation the rehabilitation program? |  | \| Paper Nr. 1 \| \| --- \|  \|  \| \| --- \| | \| Offer them to share, but they don’t have to \| \| --- \|  \|  \| \| --- \| |
| Barriers | What were the reasons against participating in the rehabilitation program? | \| Were you and your parents sure from the start that you would participate, or were you unsure at first? \| \| --- \| |  |  |
| \| Expectations \| \| --- \|  \|  \| \| --- \| | What have you planned for the rehabilitation program? |  | Paper Nr. 2 | Possibly omit the writing |
| \| Wishes/Satisfaction \| \| --- \|  \|  \| \| --- \| | \| What do you particularly like about the rehabilitation program / what do you enjoy most? \| \| --- \| |  |  | Possibly omit if already said |
|  | What don’t you enjoy? |  |  |  |
|  | \| What would you like to change about the rehabilitation program? \| \| --- \| |  |  | If you imagine you are at “Wishful Thinking”  E.g. about the daily schedule, food, times, games |
| \| Role of Parents \| \| --- \|  \|  \| \| --- \| | \| Your parents have been / are with you the whole time and are often involved.  How do you feel about your parents being here? \| \| --- \| |  | Smileys |  |
|  |  | \| When did it bother you that your parents were involved?  Can you name any examples? \| \| --- \| |  | \| E.g. cooking, therapy sessions, weighing, intake interview \| \| --- \|  \|  \| \| --- \| |
|  |  | What would you wish for regarding your parents' role during the rehabilitation program? |  | What would be some other ways of involving parents from the children’s perspective?  Possibly omit |
| \| Overall, which smiley would you give the rehabilitation program? \| \| --- \| | | | | |
| **Theme: Daily Life with the Program and Long-Term Changes** | | | | |
| \| Outpatient Approach in General \| \| --- \|  \|  \| \| --- \| | \| During the entire rehabilitation program, you were allowed to go home every day and sleep there, go to school, etc.  How do you feel about that? \| \| --- \| |  |  | \| E.g. hobbies, seeing friends, leaving school earlier  Disruptive factors; school; renunciation \| \| --- \| |
|  |  | \| Would you sometimes rather sleep here? \| \| --- \| \|  \| |  |  |
|  |  | Would you maybe have liked to be away from home for the entire 4 weeks? |  |  |
| \| Changes in Daily Life through the Program \| \| --- \|  \|  \| \| --- \| | \| What has changed in your daily routine since you’ve been participating in the program, and how did you feel about it? \| \| --- \| |  |  | \| For example, hobbies, seeing friends, leaving school earlier \| \| --- \|  \| Possibly omit \| \| --- \| |
|  |  | \| Is it tiring for you to focus on participating in the rehabilitation program while also doing other things in your daily life, like school or meeting friends? \| \| --- \| | \|  \| \| --- \| | \| Possibly rephrase \| \| --- \|  \| Possibly omit \| \| --- \|  \|  \| \| --- \| |
| \| Long-Term Transfer to Daily Life \| \| --- \|  \|  \| \| --- \| | \| You learn a lot here. What do you think you will do differently after the rehabilitation program than you did before? \| \| --- \| |  |  | \| E.g. in daily life \| \| --- \|  \| CAVE! CLICHÉS: Hobbies? Movement? Eating? \| \| --- \| |
|  |  | \| Do you feel you can continue this in the long run or do you think it might be difficult? \| \| --- \| | Smileys | Explanation: By long-term we mean “over a longer period of time” |
| Interaction of children in the rehabilitation program with other children outside the program | When you tell other people why you are doing this rehabilitation program, what do you say/tell them? |  | Paper Nr. 3 | Listen carefully to the differences in the explanations, and possibly revisit them and ask for others' opinions. |
|  |  | And have you ever done this?  Why not? | Smileys |  |
|  |  | Do you think other children understand why you are going to the rehabilitation program? | Smileys |  |
| **Theme: Emotions, Perception, Body Feeling** | | | | |
| \| Emotions of the Children and Adolescents with and since the Program \| \| --- \|  \|  \| \| --- \| | \| Great, thank you.  Now a little about you and how you’ve felt. We people often feel very different. Happy, sad, angry, scared, surprised, and so on. How do you feel when you think about going to the rehabilitation program / while you are in the rehabilitation program? \| \| --- \| |  | \| Paper Nr. 4 \| \| --- \|  \|  \| \| --- \| |  |
|  |  | \| If you like, can you describe your feelings again? \| \| --- \| |  |  |
| \| Body Feelings of the Children and Adolescents \| \| --- \| | \| How do you feel in your body? \| \| --- \| |  | Paper Nr. 5 |  |
|  |  | What does “feeling comfortable” and “feeling uncomfortable” mean to you? |  |  |
|  |  | Have these feelings changed since the rehabilitation program? |  |  |

# Parent Interview Guide

| Main Question | Follow-up Questions | Remarks |
| --- | --- | --- |
| **Icebreaker / Introduction** | | |
| Your child is participating in the outpatient rehabilitation program. You are more or less a participant as well.  What was your "path" to the outpatient rehab program? |  |  |
|  | Can you describe what motivated you to participate? |  |
| **Main Part** | | |
| **Theme: Rehabilitation Program** | | |
| You have now had time to gain some experience with the rehab program.  How do you feel about the program so far? |  | Feelings: e.g., happy, burden lifted, liberated, dissatisfied, unhappy, etc.  How does participation work?:  Are you happy when your child goes to the program or do you get a stomach ache? |
|  | How do you feel about the decision to participate in the program? |  |
|  | How do you think participation works? |  |
|  | How satisfied are you with the program? |  |
|  | What have you liked so far? |  |
|  | And what haven't you liked so far? |  |
| What were your expectations going into the program? |  |  |
|  | To what extent have they been met or not? |  |
| How do you see your role as a parent in this whole process? |  |  |
|  | What wishes do you have or did you have regarding your role? |  |
|  | How does your child perceive your role in the program? |  |
|  | What conflicts have you had with your child (regarding your role)? |  |
| The rehabilitation program is an outpatient procedure.  What do you think about it? |  | E.g. comparison: your child would remain permanently in a ward |
|  | What advantages does the outpatient program have for you compared to an inpatient procedure? |  |
|  | What disadvantages does the outpatient program have for you compared to an inpatient procedure? |  |
|  | How well is it working out for you to fit the rehab program into your daily life? |  |
| What obstacles/challenges do you see in participating in an outpatient rehab program? |  | E.g. work, time, costs, school, homesickness  Possibly distinguish between personal barriers and general/systemic barriers |
|  | What solutions do you have for the challenges/obstacles/problems mentioned? |  |
| **Theme: Everyday Life** | | |
| If you recall the time before the rehabilitation program, what would you say were the biggest challenges/problems in the daily life of your child? |  | E.g. regarding free time activities, school attendance, mental health issues, doctor visits, etc.? |
|  | How does participation in the outpatient rehabilitation program help potentially overcome these challenges/problems in the long term? |  |
| Which aids/tools for daily life that you learned in the program can you successfully integrate into your daily routine? |  | Aids/tools: E.g. nutritional counseling/cooking course, exercise, self-esteem building |
|  | What additional support would you need to transfer what you’ve learned into everyday life? |  |
|  | Can you name any aspects that have already changed in your daily life during (and due to) participation in the rehabilitation program? | Perhaps you have actively changed habits/routines or unconsciously changed by yourself? |
| **Theme: Child** | | |
| How did you perceive your child during the rehabilitation program? |  |  |
| What (long-term) changes has the rehabilitation program already brought about in your child? |  |  |
|  | For clarification, the following areas:   - Self-confidence / self-worth? - Behavior? - Movement? - Nutrition? - Social contacts? - Happiness? | There could also theoretically be negative changes, stay open to that. |

# Health Professionals Interview Guide

| Main Question | Follow-up Questions | Remarks |
| --- | --- | --- |
| **Icebreaker / Introduction** | | |
| You are an employee at the Rehabilitation Center and actively involved in the rehabilitation program. Would you mind sharing a bit about what a typical workday for you looks like? |  |  |
|  | What makes your work in the context of the program unique? |  |
| **Main Part** | | |
| **Theme: Rehabilitation Program** | | |
| Let us talk a little about the rehabilitation program itself.  How did you get involved in the program? |  |  |
| How would you evaluate the rehabilitation program? |  |  |
| What opportunities for success do you see in this program? |  |  |
|  | What does "success" mean to you in this context? |  |
|  | Do you have any examples of success opportunities? |  |
| What resources do the children, young people, and parents need at home or within the family to implement what they've learned in the long term? |  | Resources could include financial for healthier food or sports club memberships, as well as time, emotional resources, etc. |
|  | How is it asked and ensured if the resources are available to them? |  |
|  | How are families supported in this? |  |
| What challenges do you as an employee (in general) perceive in implementing the program? |  | Challenges of any kind related to outpatient rehabilitation (application, implementation, transferring to daily life, aftercare) |
|  | How do you address these challenges? |  |
|  | What needs to change, or what possibilities exist to reduce existing challenges/barriers? |  |
|  | What challenges are there in direct connection with working with children/young people and their parents? |  |
| **Theme: Working at the Program** | | |
| Now let's move a little away from the structures and focus more on the work itself. What comes to mind when you think about what brings you joy in your work with the rehabilitation program? |  | Regarding:  Working with the rehabilitants?  With the parents? |
|  | And what brings you less joy? |  |
| What skills/abilities/traits should employees in the program have to contribute to the successful implementation of the program? |  | E.g. sensitive handling of high body weight, dealing with experiences of discrimination… |
|  | What topics/trainings.., should employees focus on more or attend, or should be offered more? |  |
| **Theme: Optimizing the Program** | | |
| A program probably always involves change. Approaches may be reconsidered, or it may be explored how patients can be supported differently.  If you think spontaneously, what would you say: How could the program be expanded or improved? |  |  |
|  | Which important aspects are missing, which the program has not yet covered? |  |
| What would you change about the program? |  |  |
|  | What could be structured differently or what changes should be made to the concept? |  |
|  | Which aspects would need to be changed to better support the patients and address the individual needs of the rehabilitants? |  |
|  | Which aspects should be changed to involve the parents more?  Or maybe even less in some areas? |  |
|  | What should be changed to allow more families and children to participate in the program? |  |

Appendix 2: Excerpts from the interview and focus group guidelines
