# Supplementary material for: Shifting perspectives on the role of parents in the rehabilitation of children with higher body weight: insights from qualitative interviews with children, parents, and professionals
Source: Front Pediatr. 2026 Mar 24;14:1797335. doi: 10.3389/fped.2026.1797335 (PMC13055526; doi:10.3389/fped.2026.1797335)
Supplement: Supplementary file 3 [file Table3.docx]

**Appendix 3: Coding framework**

|  | | **Perspectives** | | |  |
| --- | --- | --- | --- | --- | --- |
|  |  | **Children** | **Professionals** | **Parents** |  |
| **Codes**  **(deductive & inductive)** | **Parental roles** | **Motivator** | | | **Expectations vs. perception** |
|  |  | **Active participant** | | |  |
|  |  | **Passive companion** | | |  |
|  |  | **Oppositional authority** | |  |  |
|  |  | **Regulating authority** |  | **Regulating authority** |  |
|  |  | **Mediator** |  |  |  |
|  |  | **Person of trust** |  |  |  |
|  |  |  | **Responsible person** | |  |
|  |  |  | **Role model** | |  |
|  |  |  |  | **Anticipated Role** |  |
|  | *Parent-child-relationship* |  |  |  |  |
|  | *Challenges* |  |  |  |  |
|  | *(Need for) external support* |  |  |  |  |
