# Supplementary material for: Shifting perspectives on the role of parents in the rehabilitation of children with higher body weight: insights from qualitative interviews with children, parents, and professionals
Source: Front Pediatr. 2026 Mar 24;14:1797335. doi: 10.3389/fped.2026.1797335 (PMC13055526; doi:10.3389/fped.2026.1797335)
Supplement: Supplementary file 4 [file Table4.docx]

Apppendix 4: Sociodemographic data of interview partners

| **Sociodemographic data** | |
| --- | --- |
| **Children and Adolescents**  Focus Groups  Age  Gender  Schooltype  Having siblings  living with siblings  *living arrangements*  with parents  with mother  with father  change between parents | *n* = 35  *n* = 6  Average = 12 years, Median = 12 years (Range: 7 - 17)  Female: *n* = 17 (49 %); Male: *n* = 16 (46%); Divers: *n* = 1 (3 %);  Not Specified: *n* = 1 (3 %)  *Primary school n =16 (46%), grammar school n = 5 (14%), comprehensive school n = 10 (28%), other types of school n = 2 (6 %)*  *n = 28 (80%)*  *n = 27 (77%)*  *n = 23 (66%)*  *n = 8 (23%)*  *n = 1 (2%)*  *n = 3 (9%)* |
| **Health Professionals**  Age  Gender  Specialization  Duration of employment in rehabilitation center | *n* = 19  Average = 42 years, Median = 41 years (Range: 23 - 60)  Female: *n* = 15 (79%); Male: *n* = 4 (21%)  Nurses: *n* = 3 (16%)  Doctors: *n* = 2 (11 %)  Psychotherapy: *n* = 4 (21 %)  Sport therapy: *n* = 2 (11 %)  Physiotherapy: *n* = 1 (5 %)  Gymnastic Teacher: *n* = 1 (5 %)  Student Health Management: *n* = 1 (5 %)  Dietitians / Nutritionist: *n* = 4 (21 %)  Social worker: *n* = 1 (5 %)  Average = 6 years; Median = 4 (Range: 1 – 16) |
| **Parents**  Age  Gender  *Family status*  Married  in a relationship  divorced  separated  *Relationship to the child*  parent | *n* = 19  Average = 44 years; Median = 43 years (Range: 40 - 54)  Female: **n** = 14 (74 %); Male: *n* = 5 (26 %)  *n* = 14 (74%)  *n* = 1 (5%)  *n* = 1 (5%)  *n* = 3 (16%)  *n* = 19 (100%) |

Apppendix 4: Sociodemographic data of interview partners
